# μOrgano: A Lego^®^-Like Plug & Play System for Modular Multi-Organ-Chips

**DOI:** 10.1371/journal.pone.0139587

**Published:** 2015-10-06

**Authors:** Peter Loskill, Sivan G. Marcus, Anurag Mathur, Willie Mae Reese, Kevin E. Healy

**Affiliations:** 1 Department of Bioengineering, University of California at Berkeley, Berkeley, California, United States of America; 2 Department of Materials Science and Engineering, University of California at Berkeley, Berkeley, California, United States of America; 3 California Institute for Quantitative Biosciences (QB3), University of California at Berkeley, Berkeley, California, United States of America; Georgia Regents University, UNITED STATES

## Abstract

Human organ-on-a-chip systems for drug screening have evolved as feasible alternatives to animal models, which are unreliable, expensive, and at times erroneous. While chips featuring single organs can be of great use for both pharmaceutical testing and basic organ-level studies, the huge potential of the organ-on-a-chip technology is revealed by connecting multiple organs on one chip to create a single integrated system for sophisticated fundamental biological studies and devising therapies for disease. Furthermore, since most organ-on-a-chip systems require special protocols with organ-specific media for the differentiation and maturation of the tissues, multi-organ systems will need to be temporally customizable and flexible in terms of the time point of connection of the individual organ units. We present a customizable Lego^®^-like plug & play system, μOrgano, which enables initial individual culture of single organ-on-a-chip systems and subsequent connection to create integrated multi-organ microphysiological systems. As a proof of concept, the μOrgano system was used to connect multiple heart chips in series with excellent cell viability and spontaneously physiological beat rates.

## Introduction

Drug research and development is an inefficient, lengthy, and expensive process [1]. Although non-human animal models have contributed significantly to pre-clinical drug development, a fundamental problem with the current process is that animal models cannot adequately represent human biology and disease states, leading to inaccurate conclusions from data obtained using these models. Recently, human microphysiological systems (MPSs) have evolved from a conceptual idea to a feasible alternative for animal models [1–6]. Various promising MPSs, also referred to as ‘organ-on-a-chip’ systems, have been introduced in recent years such as models of cardiac [7], pulmonary [8], hepatic [9,10], gut [11], renal [12], osseous [13], mammary [14], and vascular tissues [15]. While these chips featuring single organs can be of great use for both pharmaceutical testing and basic organ-level studies, the huge potential of the organ-on-a-chip technology is revealed by connecting multiple organs on one chip to create a single integrated MPS for sophisticated fundamental biological studies and devising cures for disease [16–21]. These multi-organ-chips or “human-on-a-chip” systems have the potential to also capture metabolite toxicity, off-target toxicity, and additional side effects in addition to the direct effects of compounds on a target tissue. Additionally, drug toxicity, efficacy, and efficiency *in vivo* are not only affected by the metabolism of drug compounds, but also by further complex processes such as absorption and elimination (summarized as ADME processes) [22]. Multi-organ-chips have the capacity to mimic these effects and thus provide a more clinically relevant platform with the highest predictive capability.

The realisation of multi-organ-chips faces a variety of engineering challenges, among them the fluidic control of mL and μL volumes or the maintenance and control of coupled organ systems [23]. First, promising approaches have already been introduced in recent years, consisting of a (micro)fluidic circulation connecting cell compartments of various geometries [16,19,24]. One major limitation of these systems, however, is that they contain static circulation architecture, meaning the individual organs are connected with a permanent geometry. Yet, the differentiation, formation, equilibration, and maturation of the individual tissues require in many cases special protocols with organ-specific media or small molecules. This can extend to multiple weeks in case of in-chip differentiation of either human embryonic stem cells (hESC) or induced pluripotent stem cells (hiPSCs). Additionally, the variability among batches and cell lines with regards to differentiation efficiency and tissue formation, significantly decreases the success rate of the overall system in case of permanent connection between cell compartments for stem cell based systems [25].

**Fig 1:**
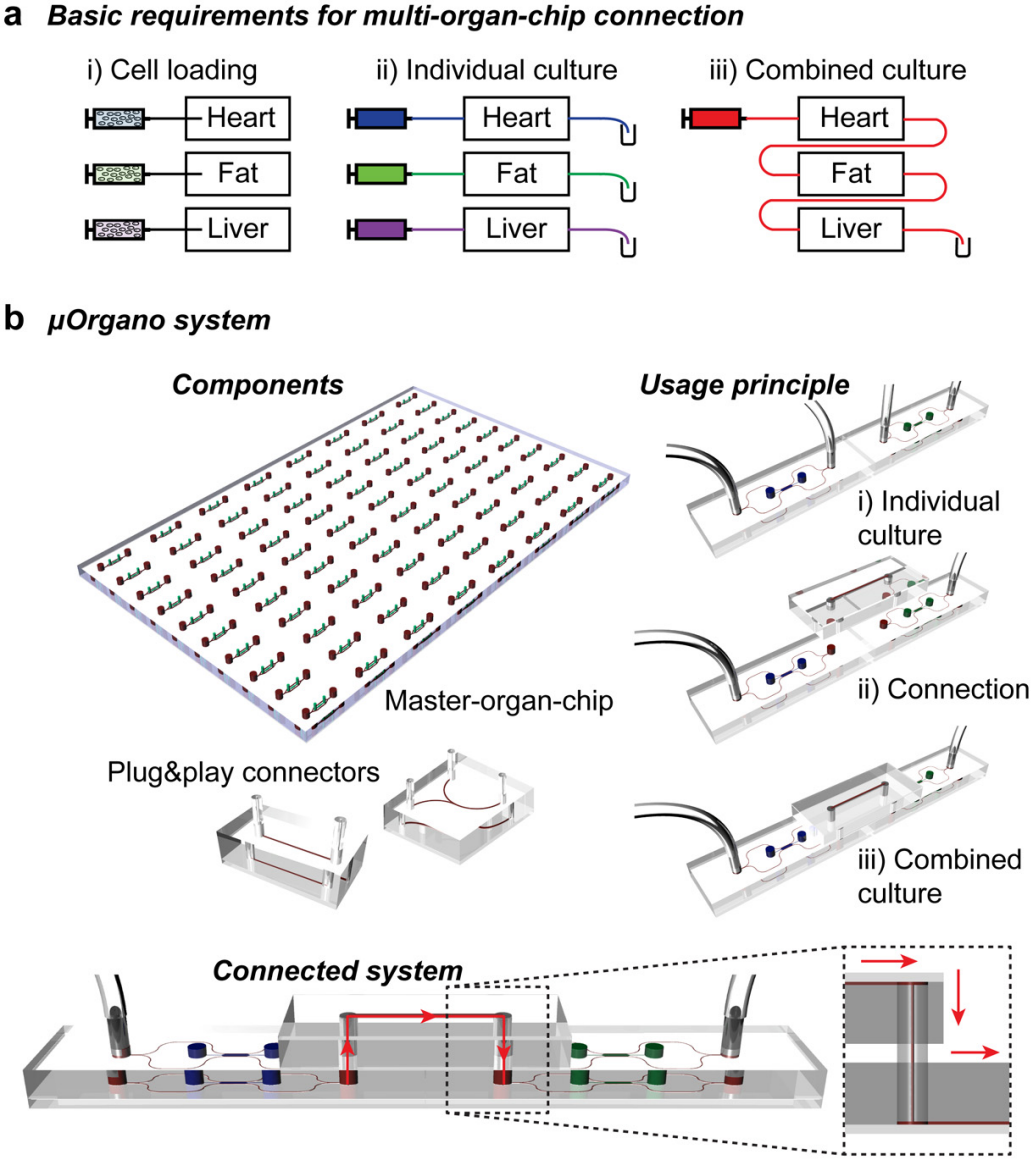
Challenges and solution for multi-organ-systems. **a**) General requirements for multi-organ-chips: i) initial separate loading of the respective cells; ii) individual culture for differentiation, formation, equilibration, and maturation of the tissues; and, iii) combined culture for drug screening purposes. **b**) Underlying concept of the μOrgano system: Schematics depicting the basic μOrgano components: the master-organ-chip and exemplary plug & play connectors. Conceptual idea of the usage principle of the μOrgano system for the connection of two MPSs in series via a simple linear channel connector with a close-up of the connected system highlighting the resulting media flow.

Here, we present a customizable Lego^®^-like system that addresses the aforementioned issues and enables a fluidic control of μL volumes. Multiple plug & play or Lego^®^-like approaches exist for various applications such as biochemical synthesis [26] and analysis [27], mixing and reaction systems [28], on-chip cell concentration [29], detection of bacterial pathogens [30], or macroscale 3D systems based on standardized discrete elements [31]. The system we present here, from now on referred to as μOrgano, is specifically designed to connect multiple organ-on-a-chip (μ-organs) systems into multi-organ-chips. The μOrgano is a plug & play system that allows for: i) separate loading of different cell types; ii) temporal control of individual culture of cells for differentiation and tissue development; and, iii) subsequent temporal control of fluidic connections of the individual tissues (Fig 1a). This paper focuses on the development of the μOrgano system with a proof of concept using MPSs incorporating hiPSC-derived cardiomyocytes (hiPSC-CMs).

## Materials and Methods

### Fabrication of Connectors

To create the connectors, 45 μm high and wide square channel structures were patterned with SU8 3050 photoresist (MicroChem Corp, Newton, MA) onto silicon wafers (University Wafer, Boston, MA) according to the manufacturer’s data sheets. Subsequently, 1.5 mm high posts (diameter 2 mm) were patterned at the designated locations for in- and outlet ports. Similar to a protocol by Bian *et al*. [32], six layers of 250 μm thick SU8 100 photoresist (MicroChem Corp, Westborough, MA) were spin-coated (10 s at 500 rpm + 30 s at 1000 rpm) on top of the channel structures. Following each individual spin-coating step, the wafers were baked for 15 min at 65°C and 2 hours at 95°C. The entire coating process was then finalized by a soft bake at 95°C for 12 hours. The patterning was achieved by exposing the coated wafers to 33 mW/cm^2^ UV light using a mask aligner (Hybralign Series 200, OAI, San Jose, CA) for a total of 4 min (60 s exposures interrupted by 2 min cool down times). The exposed wafers were then developed, baked for 24 hours at 40°C, and functionalized using a Tridecafluoro–1,1,2,2-Tetrahydrooctyl)Trichlorosilane (Gelest, Morrisville, PA). By performing exclusion molding on these wafers, connectors with prefabricated in- and outlet holes were fabricated. Briefly, uncured polydimethylsiloxane (PDMS, Sylgard 184, Down Corning, Midland, MI)– 1:10 w/w ratio of curing agent to prepolymer—was poured onto the wafer and subsequently covered with a mylar sheet, which was clamped onto the wafer using a glass slide. After overnight curing at 60°C, the mold was peeled from both the wafer and the mylar sheet. The molded connectors were then cut into individual modules, which were then bonded to microscope glass slides by exposing them to oxygen plasma (Plasma Equipment Technical Services, Livermore, CA) at 60 W for 20 s and subsequent baking at 60°C for 3 h. Glass capillaries (Micro Bore Tubings, Accu-Glass, St. Louis, MO) were manually cut using a capillary cutting stone (Hampton Research, Aliso Viejo, CA) and subsequently boiled in Milli-Q water for 1 h in order to dull the edges and prevent damaging the PDMS devices. Following a cleaning step using 1 M sodium hydroxide for 1 h at room temperature, the capillaries were bonded into the in- and outlet ports of the connectors by exposing them to oxygen plasma at 60 W for 20 s and subsequent baking at 60°C for 3 h.

### Fabrication of MPSs

The cardiac MPSs were fabricated via a two-step photolithography process as described previously [7]. Briefly, in the first step, 2 μm high ‘‘endothelial-like” barriers and a weir gap were patterned via UV lithography using SU–8 2001 photoresist (MicroChem Corp) a first step. In the second step, the 35 μm high media and cell culture channels were fabricated using SU–8 3025 (MicroChem Corp). The patterned wafers were then baked, and coated with trichloro-1H, 1H, 2H, 2H-perfluorooctylsilane (FOTS, Gelest, PA, USA). MPSs were replica molded by pouring uncured PDMS (Sylgard 184, Down Corning, Midland, MI)– 1:10 w/w ratio of curing agent to prepolymer—onto the master wafer and cured overnight at 60°C. PDMS devices were aligned and bonded to microscope glass slides after exposure to oxygen plasma (Plasma Equipment Technical Services, Livermore, CA) at 60 W for 20 s. To stabilize bonding, the devices were subsequently baked at 60°C for 3 h.

### Connection of MPSs

Before usage, the sterility of the connectors had to be ensured. Therefore, the modules were flushed for 30 min with 70% ethanol, subsequently washed with PBS for 30 min using a PhD Ultra syringe pump, and stored under sterile conditions. Immediately prior to the experiments, the connectors were prefilled with the respective cell culture medium. After carefully removing the tubings necessary for the separate feeding from the out- and inlet ports of the MPSs, the prefilled connectors were inserted into the respective ports under sterile conditions. The connected systems were then fed using continuous flow from a syringe pump and placed under standard cell culture conditions. To validate the bubble-free connection, we prefilled the connectors with food dye (DecACake) coloured Milli-Q water and focused a bright field microscope on a section of the media channel in the immediate proximity of the inlet of the (defined by the media flow) second device. Video microscopy data taken at the temporal onset of the media flow showed the replacement of the colourless by the coloured liquid and was then analysed for the occurrence of bubbles. To characterize the transport times necessary for the media to flow from one cell chamber to the next one, we prefabricated systems consisting of two MPSs connected via linear connectors featuring capillaries of different inner diameters (IDs). These systems were infused with food dye coloured Milli-Q water at a rate of 20 μL per hour via syringe pump and the time manually measured via visualization of the flow under a microscope. To test the reproducibility of the bifurcation, we connected multiple systems consisting of three MPSs each with bifurcation connectors. We then pumped dye coloured Milli-Q water at a rate of 20 μL per hour via syringe pump into the first MPS, collected the media from the outlet ports of the two other MPSs in neighboring reservoirs (placed in a closed humid environment next to sacrificial water containers), and determined the volumes in both of these reservoirs after 20 h.

### Loading of MPSs and tissue characterization

Cardiac tissues inside the MPSs were generated as described previously [7]. Briefly, human CMs were derived from hiPSCs via modulation of the WNT pathway, using an optimized directed cardiac differentiation protocol [7,33]. At day 15 of the differentiation process, we dissociated the beating CMs using a singularization protocol introduced by Zhu *et al*. [34] The cell chambers of the MPSs were pre-coated with fibronectin (20 μg/mL in PBS) for 1 h at 37°C subsequent to hydrophilizing and sterilizing them for 3 minutes at 180 W using O2 plasma (PETS Reactive Ion Etcher). Cells were loaded into the MPS by applying 100–200 μL of a cell solution (4–5 million cells/mL) to the cell inlet port and employing a negative pressure at the outlet ports utilizing a PhD Ultra syringe pump (Harvard Apparatus). The loaded devices were then fed using a syringe pump with a continuous flow of EB20 media (Knockout DMEM supplemented with 20% fetal bovine serum (FBS), 2 mM L-glutamine, 1× MEM non-essential amino acids (MEM-NEAA), 400 nM 2-mercaptoethanol) (Life Technologies). For the first 24 h the media was supplemented with 10 μM Y–27632 (BioVision). After successful formation of a robust tissue with homogeneous beating behavior, the feeding was continued with serum-free media (RPMI 1640 containing B27 with insulin supplement). The loading of 3T3 fibroblasts into the MPSs was performed analogously with the only difference being the feeding media, which consisted of DMEM (Invitrogen) supplemented with 10% FBS and 1% Pen/Strep. To characterize viability, the connected MPSs were washed with sterile PBS (Corning) via syringe pump infusion at a rate of 5 μL/min for 15 minutes. Following this, cells were stained using a solution of 2 μM Calcein^®^, AM and 4 μM ethidium homodimer–1 (Life Technologies) in sterile PBS, infused via pump at 5 μL/min for 45 minutes. After staining, devices were imaged via fluorescent microscopy (Nikon Eclipse TE300, Nikon, Tokyo, Japan). To characterize functionality of the cardiac tissues, bright field movies of the beating tissues inside the MPSs were taken using the Nikon Eclipse TE300 microscope fitted with a “QICAM Fast” camera (QImaging, Surrey, BC, Canada). These movies were subsequently analysed using our custom motion tracking software (available under a GNU license at http://gladstone.ucsf.edu/46749d811/; Matlab-based (MathWorks, Natick, MA)) utilizing parallel computing on all cores of a 12-core Mac Pro (Apple, Cupertino, CA) as described previously [35]. The block matching based software quantifies the beating motion and outputs motion kinetics with characteristic beating and relaxation peaks allowing for quantification of parameters such as beat rate.

## Results

The basic building blocks of the μOrgano are: i) a master-organ-chip; and, ii) plug & play connectors. The master-organ-chip consists of a grid-like arrangement of individual MPSs (Fig 1b). These MPSs can be a custom combination of different organ-on-a-chip systems including, but not limited to the previously presented systems [7–9,12,15]. The sole prerequisite for a compatible MPS is that it contains defined media inlet and outlet ports, which can be arranged on an equidistant grid. Other than this prerequisite, there are no limitations in terms of design and characteristics of the MPSs. As a proof of concept, we focused on master-organ-chips consisting of multiple units of the cardiac MPS recently introduced by Mathur *et al*. [7]. This MPS consists of a central cell chamber, two adjacent media channels, and arrays of connecting microchannels. This design creates purely diffusive transport of media compounds between the media channels and the cell chamber, with diffusion properties similar to the endothelial barrier present in the human *in vivo* vasculature.

The plug & play connectors consist of small microfluidic devices featuring channel structures, and inlets and outlets equipped with open cylinders (Fig 1b). The length of the cylinder corresponds to the combined thickness of master-organ-chip and connectors. These connectors can be “plugged” into the in- and outlet ports of the master-organ-chip and thereby used to connect two (or more) individual MPS units (Fig 1b). The channel structures can range from simple linear channels (length matching n x grid constant or n x √2 x grid constant) connecting two neighbouring MPS units to more complex structures such as bifurcations (Fig 1b), which split the flow to two different MPS units. Bifurcations with different channel widths allows for a controlled distribution of dissimilar flows into different MPSs. The combination of multiple bifurcations and/or linear connectors enables complex systems providing a further step towards the recapitulation of the *in vivo* circulation. In general, a toolbox of connectors with various structures enables the creation of customized circulation architectures.

**Fig 2:**
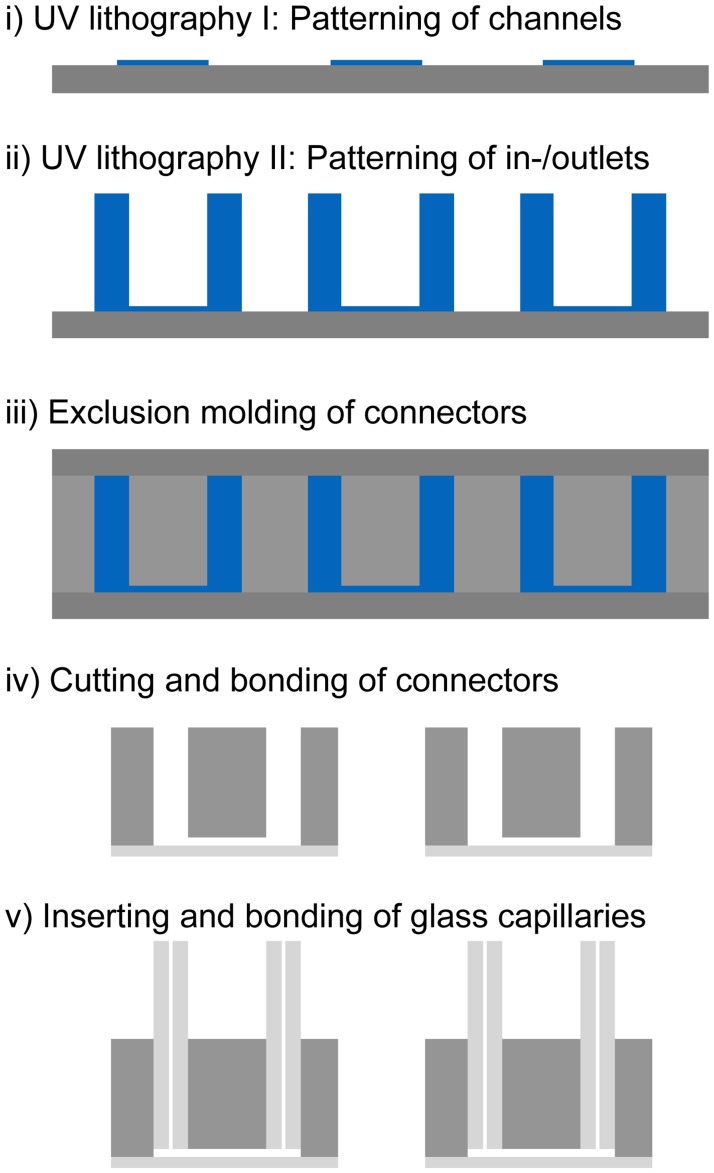
Fabrication of μOrgano building blocks. Schematic protocol for the fabrication of connectors (and MPSs) with precise in- and outlet positions via multi step UV-lithography: i) microscopic channel structures are patterned in photoresist using UV lithography; ii) macroscopic in- and outlets are patterned as pillars on top of the microscopic channel structures using a second UV lithography step; iii) microfluidic PDMS devices are fabricated with predefined in- and outlets via exclusion molding; iv) PDMS connectors are cut and bonded to pre-cut microscope slides; and, v) glass capillaries are inserted and bonded into the in- and outlets of the connectors.

The fabrication and practical implementation of the μOrgano system provided three major challenges: i) precise and reproducible in- and outlet positions were necessary to allow for a plug & play connection; ii) the dead volume inside the connectors needed to be minimized in order to have physiological transport times for the media to travel from one MPS to the next one; and, iii) the insertion of the plug & play connectors must result in a sealed and bubble free system. To create in- and outlet ports in the connectors with precise spatial orientation and reproducible straight vertical channels, manual punching using biopsy punches commonly used for fabrication of microfluidic devices was not feasible. Previous attempts to directly fabricate ports placed posts manually on the master before the replica molding [36] or utilized double casting approaches with either manual coring of the intermediate molds [37] or a combination of milling and hot-embossing [38]. We avoided any type of manual handling as well as double casting and thereby achieved a higher precision and spatial resolution by employing a combination of multi-step UV lithography and exclusion molding of the PDMS devices as depicted in Fig 2: First, we patterned microscale channel structures of appropriate geometries for the different types of connectors (Linear, bifurcation,…). Subsequently, macroscale posts as templates for the in-and outlet ports were patterned precisely at the respective ends of the channels.

To minimize the dead volume inside the connectors, it was essential to reduce the volume of the tubes in the in- and outlet ports, since these were the sole components not created by microfabrication. This requirement is not sufficiently met by stainless steel catheter couplers (typically 1.3–3 μL / 10 mm tube) commonly utilized for microfluidic devices. A useful alternative is the use of glass capillaries (Micro Bore Tubings, Accu-Glass, St. Louis, MO) with small IDs of 50 ~ 200 μm (≈ 0.02 ~ 0.3 μL/10 mm tube). These capillaries were bonded in the connector ports resulting in Lego^®^-type connector modules with small volumes. The resulting volume of the connectors is defined by the volume of the connecting channel (≈ 0.026 μl for in-series modules), the volume of the capillaries (≈ 0.016 μL for capillaries (8 mm) with 50 μm ID; ≈ 0.2 μL in case of 150 μm ID), and the volumes between the end of the capillaries and the glass slide (A side effect of the finite roughness of the ends of the manually cut capillaries is a spacing between capillaries and glass slides and thereby a prevention of occlusion of the channels). To obtain the entire “inter-MPS volume”, the channel volume of two halves of the MPS (≈ 0.108 μL total for the cardiac MPS) has to be taken into account as well. The actual choice of ID for the capillaries requires a balancing of minimization of dead volume and hydraulic resistance. Hydraulic resistances can be obtained using $R_{\text{circ}}=\frac{8\mu }{\pi }\frac{L}{R^{4}}$ for channels with circular crossections (length L, radius R) and $R_{\text{rec}}=\frac{12\mu L}{\left(\frac{w}{h}-0.63\right)h^{4}}$ for channels with rectangular crossection (length L, width w > height h) by assuming a viscosity ***μ* = 0.78** mPa s (Dulbecco’s modified eagle medium (DMEM) with supplements at 37°C) [39]. As detailed in Table 1, feeding two connected MPSs with a typical flow rate of 20 μL/h causes a back pressure of approximately 10 mbar when using capillaries with 50 μm ID and about 6 mbar with 150 μm ID. In the case of larger systems with ten MPSs in series, the back pressure can reach up to ≈ 80 mbar and ≈ 40 mbar respectively. The resulting values, however, provide no problems for typically used macroscopic pumps as well as most micropumps [40,41], and also allow for the utilization of gravity feeding (approximately 6–80 cm height difference).

**Table 1:** Hydraulic resistance and back pressure occurring at a typical feeding rate of 20 μL / h for individual MPSs, linear connectors, and connected systems.

	Capillary ID (μm)	Hydraulic resistance (mbar / (μL/h))	Back pressure (mbar) (for 20 μL/h)
MPS		0.075	1.5
Linear connector	50	0.363	7.3
Linear connector	150	0.140	2.8
2 connected MPSs	50	0.513	10.3
2 connected MPSs	150	0.290	5.8
5 connected MPSs	50	1.827	36.5
5 connected MPSs	150	0.934	18.7
10 connected MPSs	50	4.017	80.3
10 connected MPSs	150	2.008	40.2

**Fig 3:**
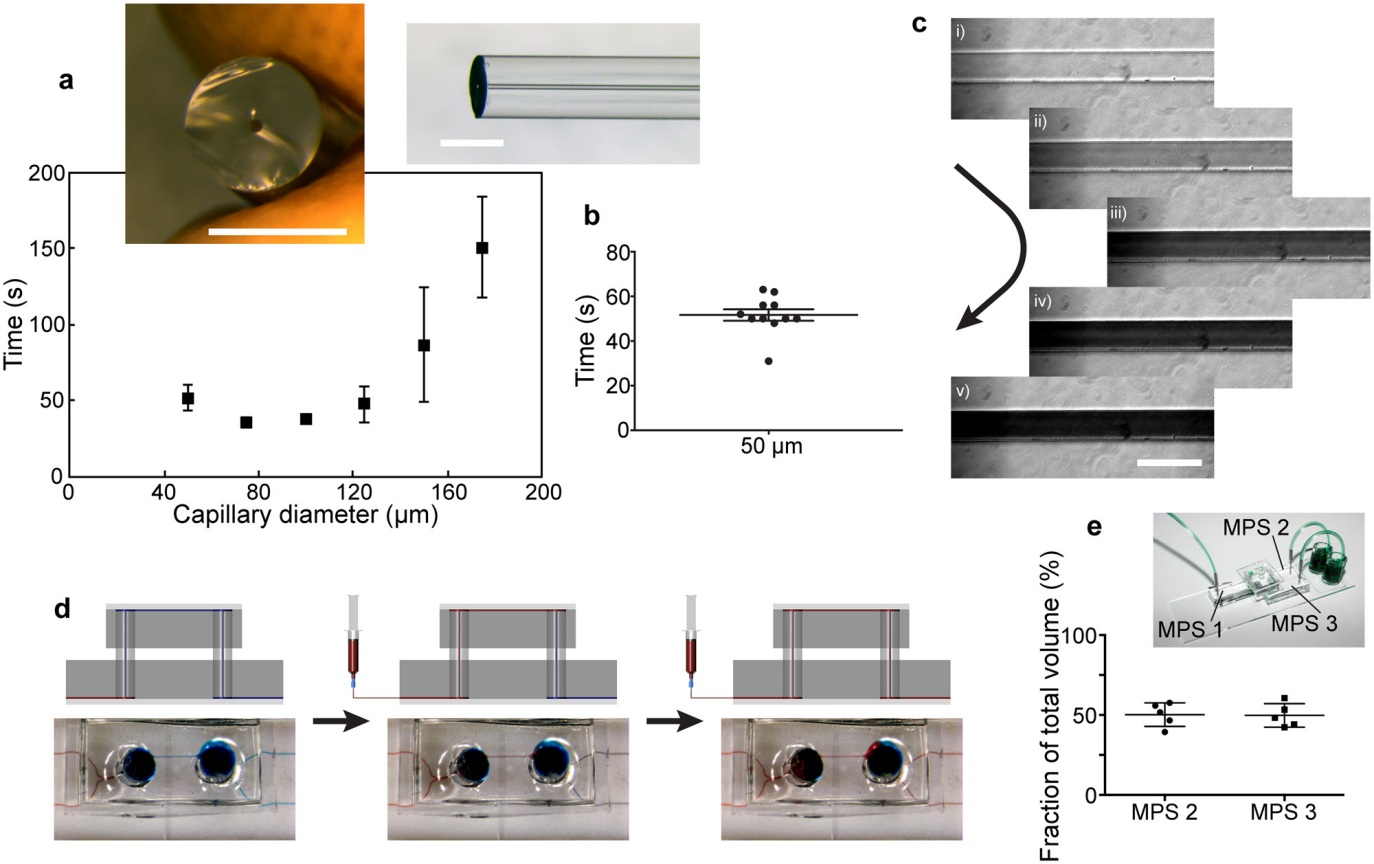
Characterization of μOrgano building blocks. **a**) Transition time of the interface of a liquid advancing through a system of two MPSs and a linear connector. The time necessary to advance from the cell chamber in MPS 1 to the cell chamber in MPS 2 is plotted versus the inner diameters of the glass capillaries in the respective systems. Insets show pictures of the respective glass capillaries (scale bars = 2 mm). **b**) Scatter plot of the transition times for ten independent systems connected by the same type of connectors featuring 50 μm ID capillaries. **c**) Time series of microscopy images from a channel section in the proximity of the inlet of the second MPS initially filled with clear water. The continuous transition occurring after connection to a MPS filled with coloured water using a food dye reveals the bubble less connection ability of the system (scale bar = 100 μm). **d**) Time series of pictures showing two MPSs connected by a linear connector whereby MPS 1 is prefilled with red dyed water, and MPS 2 and the connector with blue dyed water. Pumping red dyed water into MPS 1 leads to the replacement of the blue dyed water in both the connector and MPS 2 without the occurrence of leakage. **e**) Volume flown through MPS 2 (left; in flow direction) and MPS 3 (right) plotted as percentage of the total volume after connection to MPS 1 via a bifurcation connector.

To measure transport times of the media travelling from one MPS to the next one, we connected pairs of cardiac MPSs using a linear connector. By pumping dyed Milli-Q water through the system (20 μL/h) and measuring the time necessary to travel from the cell chamber in MPS 1 to the one in MPS 2, we confirmed physiological transport times in the range of ~ 50 s to 150 s for capillaries with various IDs (Fig 3a), representing transported volumes in the range of ≈ 0.3–0.8 μL respectively. These experimental values indicate that the previously unknown average spacing between the end of the glass capillaries and the microscope slides are in the range of 10–20 μm. The reproducibility of the connection step was validated by repeating the measurement in ten independent systems, which were connected with connectors featuring capillaries with 50 μm ID, revealing only small variations in the transport times (Fig 3b). These variations were partly due to slight differences in capillary lengths leading to differences in the inter-MPS volume. However, despite these variations the physiological character of transport times is ensured. Additionally, a large-scale automatized fabrication of the capillaries with precise length control will significantly reduce this variability.

A sealed and bubble free system was achieved by both bonding of the capillaries into the connectors, and prefilling of the connectors with the required media before inserting them into the master-organ-chip. Thereby, the media flow after connection takes place without occurrence of air bubbles (Fig 3c; S1 Movie) or leakage (Fig 3d). To test the performance of the bifurcation connectors in terms of reproducibility and evenness of flow splitting, we pumped dye coloured Milli-Q water into MPSs, which were connected to two MPSs each using bifurcations. Measuring the liquid volumes in the respective outlet ports revealed an even splitting of the input flow (Fig 3e), whereby the slight variations can again be traced back to small differences in capillary lengths due to the manual cutting process.

**Fig 4:**
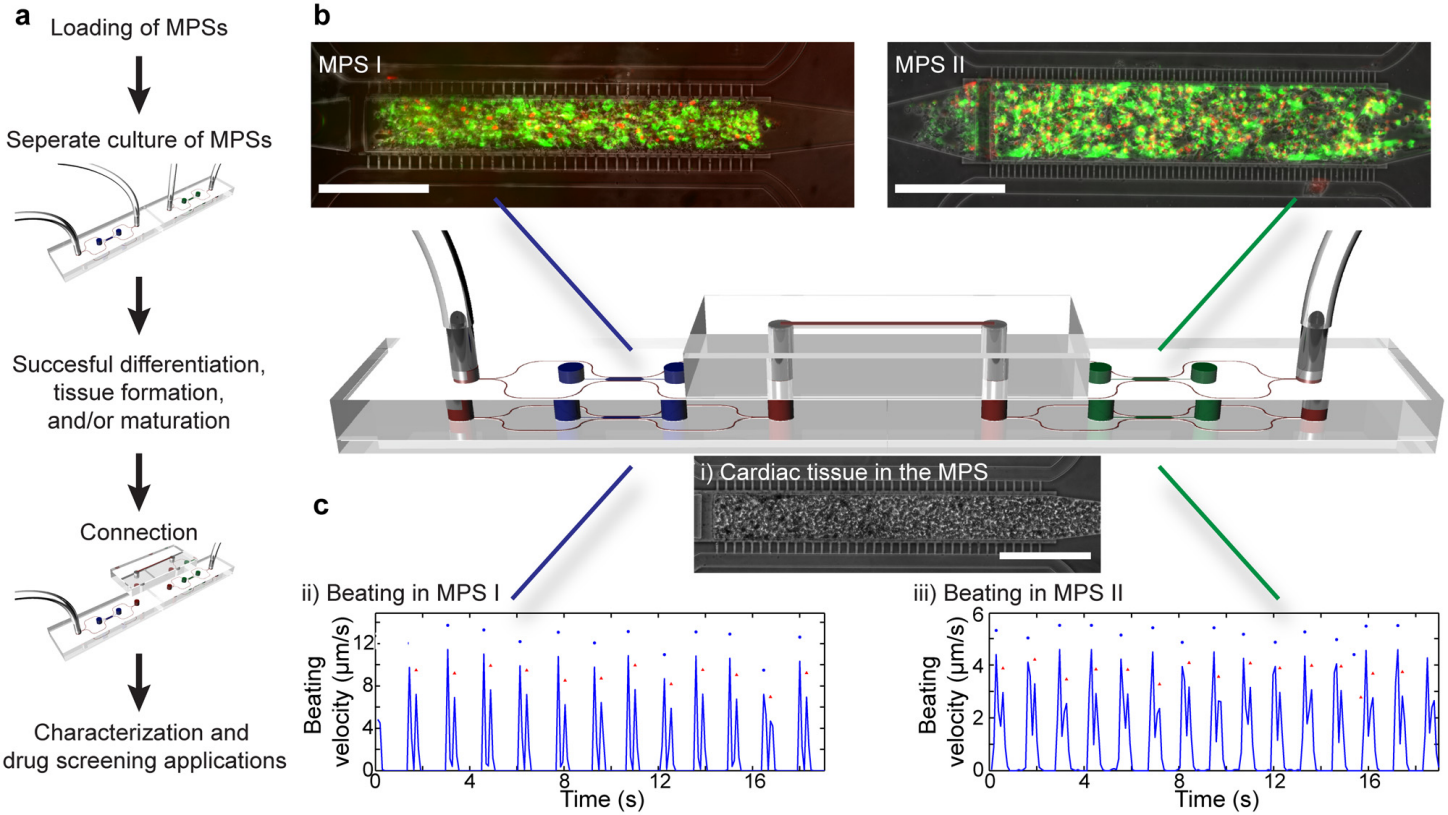
Proof of concept of the μOrgano system. **a**) General procedure for biological experiments with the μOrgano system. **b**) Combined culture of two devices with 3T3 fibroblasts: Live (green) /dead (red) staining in both devices after 1 day of individual and 2 days of combined culture show that viability can be maintained. **c**) In-series culture of two heart-on-a-chip devices: tracings of the beating motion of cardiac tissue formed by hiPSC-cardiomyocytes—i) optical microscopy image—in two connected MPSs (ii) MPS 1; iii) MPS 2). The analysis using computational motion tracking reveals that a physiological phenotype is retained and individual cardiac devices beat with distinct frequencies. (scale bars = 200 μm).

The use of the μOrgano system for cell culture requires sterility of the system in order to prevent contamination. The biocompatible PDMS/glass hybrid modules allow for standard sterilization methods. As a proof of principle for the applicability for cell culture systems, we injected 3T3 fibroblasts into two MPSs and cultured them separately for 48 h. After connecting them with a linear connector and subsequent in series-culture for another 72 h, we performed a live/dead stain. Fluorescent imaging of the stained MPSs (Fig 4a) revealed the viability of the cells in both of the connected MPSs, confirming the capability of the μOrgano system to keep cells viable and thereby validating its general applicability for cell culture systems. To validate the ability of the μOrgano system to connect organ-on-a-chip devices while retaining their functionality, we generated functional human cardiac tissue in two MPSs by injecting hiPSC derived cardiomyocytes in two cardiac MPSs [7]. The two devices were then fed independently for 3 days with a serum containing media. To ensure cardiac tissue formation, the media was supplemented with an inhibitor of Rho-associated, coiled-coil containing protein kinase (ROCK) for the first 24 h after loading. After 3 days, robust tissues were formed in each of the two MPSs. The tissues showed homogeneous beating with physiological beat rates. Subsequently, the systems were switched to a serum-free media and connected with a linear μOrgano connector. After 24 h in-series culture, using video microscopy analysis, both devices beat homogeneously at physiological beat rates validating the capability of the μOrgano system to enable the maintenance of a functional phenotype in connected heart-on-a-chip devices (Fig 4b). Although these heart-on-a-chip devices beat spontaneously at similar rates, the beating was independent from each other, indicating each device behaves as a technical replicate and therefore an array of devices can be used for high content screening during drug development.

## Discussion

The applicability of the μOrgano system for organ-on-a-chips was demonstrated by combining two heart-on-a-chips. In general, there are no theoretical limits to the overall size of the master-organ-chip as well as the number and type of MPSs in a single system. The chip, moreover, could in principle be either a baseplate on which individual MPSs are plugged in or a prefabricated static system. To enhance practical use, however, static master-organ-chips matching the dimensions of standard well plates might be beneficial. Thereby, the compatibility with microplate readers and other standard laboratory equipment is ensured. Standard well plate dimensions allow, for instance, more than 100 individual units of the cardiac MPS used in this study.

A major unmet challenge in the field is the development of common media [23], which will enable the application of the μOrgano system for the combination of different organs and pave the way for fundamental studies on the interactions between multiple organs and advanced drug screening experiments. Yet, already at this stage without a common media, the application of the system for the combination of multiple modules of the same organ opens up interesting studies, such as investigation of scaling effects or parallel screening of healthy and diseased systems. The temporally customizability and flexibility of the μOrgano concept in terms of the time point of connection of the individual organ units additionally breaks down barriers for future systems including MPSs that require tissue-specific treatments for differentiation, tissue formation, and maturation.

The plug & play character of the system enables customized circulations with a large degree of flexibility, which are envisioned to open up a variety of interesting applications, such as: i) the variation of the relative sizes of organs by integrating multiple replicates of one organ; ii) the examination of pathological states by adding disease-specific systems in parallel to healthy systems; and, iii) the addition of redundant MPS units in parallel to enable a bypassing of dysfunctional single units minimizing the dependency of the multi-organ system on the error-proneness of the individual MPSs. These opportunities together with the key ability of the system to combine multiple organ-chips while maintaining μL-scale volumes provide a solution for some of the important engineering challenges paving the way for multi-organ-chips [23]. Although the main application of the system is envisioned as a single use style, the connectors can also be safely taken out to break the fluidic connections if necessary and re-inserted again at a later time point.

Adding more complex plug & play connectors will evolve μOrgano system into a more advanced and potentially powerful tool and help to address further remaining challenges. For example, the integration of MEMS sensors into the connectors will enable the real time access of parameters such as pH, oxygen level, or glucose concentration at different positions in the *in vitro* circulation. Along the same line, equipping connectors with valves will allow injection of compounds or extraction of samples, and the integration of microscale pumps will permit closed circulation architectures.

In summary, we have presented a platform with the potential to create multi-organ MPSs that will have great potential in drug screening applications, as well as pharmacological and pharmacokinetic research. Salient features of the design are the precise and reproducible in- and outlet positions for a plug & play connection, minimization of dead volume inside the connectors, and a sealed and bubble free system. These developments allow systems capable of integrating initially separate individual MPSs and a subsequent combined culture maintaining tissue viability and functionality.

## Supporting Information

S1 Movie Media flow after connection. Bright field microscopy movie of a section of the media channel in the immediate proximity of the inlet of the (defined by the media flow) second device in a system of two in series-connected devices showing the replacement of a colourless by a coloured liquid without the occurrence of bubbles.
(AVI)
